# Undoped and doped wurtzite GaAs probed by polarization- and time-resolved cathodoluminescence†

**DOI:** 10.1039/d5na00206k

**Published:** 2025-04-10

**Authors:** Hung-Ling Chen, Thomas Bidaud, Andrea Scaccabarozzi, Romaric De Lépinau, Fabrice Oehler, Gilles Patriarche, Sophie Bouchoule, Jean-Christophe Harmand, Andrea Cattoni, Stéphane Collin

**Affiliations:** a Centre de Nanosciences et de Nanotechnologies (C2N), CNRS, Université Paris-Saclay Palaiseau France stephane.collin@cnrs.fr; b Institut Photovoltaïque d’Ile-de-France (IPVF) Palaiseau France

## Abstract

Nanowires (NWs) offer unique possibilities to control semiconductor heterostructures and polytypes at the nanometer scale. The crystal structure of GaAs can be switched from bulk cubic zinc blende (ZB) to the hexagonal wurtzite (WZ) phase, but the properties and doping of WZ GaAs are still poorly known. Here, we grow high-quality GaAs NWs containing large segments of pure ZB and WZ phases using self-catalyzed, vapor–liquid–solid molecular beam epitaxy. Undoped, Be-doped and Si-doped WZ GaAs are investigated by high-resolution cathodoluminescence (CL) at low temperature (10 K). The luminescence originating from the WZ region is unambiguously distinguished by its strong anisotropy, evidenced by polarimetry. In undoped GaAs, the WZ CL peak is found ∼1 meV higher than the free exciton energy in ZB. The recombination dynamics is probed by time-resolved CL and features a lifetime of 0.6 ns for exciton recombination and 1.65 ns for the free-electron-to-acceptor transition. From Be-doped NWs, we infer an ionization energy of ∼30 meV for the Be acceptor in GaAs WZ. The CL spectra broaden and redshift with increasing Be concentration due to the bandgap narrowing, following a trend similar to GaAs ZB. Si-doped WZ GaAs exhibits a low-energy CL peak (1.47 eV) attributed to the donor–acceptor pair recombination involving Si impurities. The degree of polarization of WZ luminescence decreases with increasing doping levels for both p-type and n-type. These results shed light on the properties and doping of WZ GaAs and show that time-resolved CL and CL polarimetry constitutes a powerful tool to characterize the crystal phase, local defect, transport and recombination mechanism at the nanoscale.

## Abstract 2

The WZ and ZB phases of single GaAs nanowires are probed by cathodoluminescence (CL) mapping, CL polarimetry and time-resolved CL.

## Graphical abstract

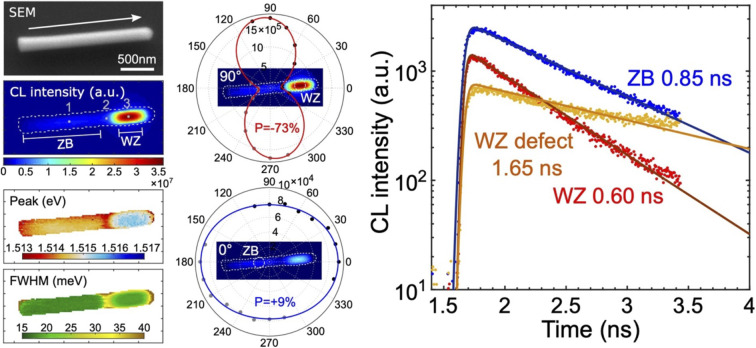

## Introduction

Nanostructures provide a unique way to control crystal phases that are not naturally found in bulk semiconductors.^1–4^ Compound semiconductors (III-phosphide and III-arsenide) usually form a cubic zinc blende (ZB) crystal structure in bulk and thin films, but they may adopt a hexagonal wurtzite (WZ) structure when grown in nanowires (NWs).^5–12^ It has been predicted that indirect bandgap semiconductors such as GaP and AlP become direct bandgap in the WZ form,^13^ enabling their use for efficient green light-emitting diodes.^14,15^ GaAs NWs are potentially interesting for opto-electronic devices such as high-speed photodetectors^16,17^ and solar cells,^18–20^ enabling the direct growth of III–V on silicon for the realization of III–V/Si tandem solar cells.^20–22^ Crystal phase switching in single NWs also provides an opportunity for implementing quantum heterostructures without varying the chemical composition^1,23^ or to control the crystal phase in other semiconductors, for example, by forming hexagonal SiGe in a shell around wurtzite GaAs nanowires.^4^ However, an uncontrolled mixture of ZB/WZ phases may degrade the device performance. Besides, the optoelectronic properties of WZ GaAs reported so far are controversial. The bandgap of WZ GaAs or low temperature exciton transition was reported to be either similar to that of ZB GaAs (within a 5 meV difference)^10,24–30^ or 20–40 meV higher.^31–34^ The discrepancies arise from the coexistence of the two crystal phases,^35–37^ strain effects^38^ and quantum confinement.^39^

A specific feature of the WZ lattice is its lower symmetry compared to the ZB lattice, resulting in different optical selection rules. The WZ lattice is described by the point group *C*_6v_ (6-fold rotation axis and 6 mirror planes parallel to the rotation axis), in which the dipole transition between the conduction band (*Γ*_7_ or *Γ*_8_) and the highest valence band (*Γ*_9_) is forbidden in the direction parallel to the WZ [0001] *c*-axis (*i.e.* the NW growth direction). Strong absorption of light perpendicular to the NW axis was observed by measuring the polarization dependence of photocurrent,^27^ photoluminescence (PL)^40–42^ or resonant Raman scattering.^25,26,43^ However, these techniques cannot provide detailed information at the nanoscale or resolve the carrier diffusion or recombination dynamics. Moreover, intentional doping in WZ GaAs is rarely explored^44,45^ while the precise control of doping is essential in functional devices. In the following, we address this issue by extending our recent results obtained for ZB GaAs nanowires to the WZ crystal phase, and we further investigate the dynamics of charges by time-resolved cathodoluminescence (CL). The present work builds upon our previous study of a series of GaAs nanowires, leveraging the spectral and spatial capabilities of CL, which enabled a quantitative determination of the density of carriers at the nanometer scale for both p-type (Be dopants) and n-type (Si and Te dopants) ZB GaAs.^46–49^

Here, we use high-resolution CL mapping combined with polarimetry and time-resolved CL (TRCL) analysis to study undoped and doped GaAs NWs containing large sections of pure WZ and ZB structures, which are known to contain no visible defects or stacking faults based on TEM characterization. We probe single nanowires in order to compare the WZ and ZB regions grown under the same conditions, with the same diameter and passivation shell made of AlGaAs. CL emission from the WZ crystal phase is unambiguously distinguished by analyzing the polarization state of light. We infer a low-temperature (10 K) excitonic transition of about 1.516 eV in undoped WZ GaAs, 1 meV higher than the free exciton energy in ZB GaAs. TRCL measurements reveal a longer lifetime for a shallow WZ defect (1.65 ns) as compared to the WZ excitonic transition (0.60 ns). We observe the bandgap narrowing (BGN) effect with increasing Be doping, suggesting a significant hole concentration in these Be-doped WZ GaAs NWs. Si-doped WZ GaAs presents a broad CL spectrum originating from Si-related donor–acceptor pair recombination.

## Nanowire growth and structure

### Selective area growth of GaAs NWs

GaAs NWs were grown by Molecular Beam Epitaxy (MBE) using the self-catalyzed vapor–liquid–solid (VLS) method on a Si (111) substrate covered with a patterned SiO_2_ mask to localize the NW growth in ordered arrays. MBE growth began with Ga predeposition, followed by the supply of As_4_ to grow GaAs NWs at 610 °C *via* the VLS method. Subsequently, Ga droplets were crystallized by closing the Ga shutter, lowering the substrate temperature to 500 °C and increasing As_4_ flux to roughly 2–3 times as used for the VLS growth. These steps were kept as identical as possible for all samples and produced GaAs NWs containing a long ZB section (1 μm) up to a WZ segment (300 nm) and terminated with a short ZB tip. For Be-doped NWs, Be flux was simply added during the VLS growth of GaAs NWs, with nominal doping concentrations of 1 × 10^18^ and 9 × 10^18^ cm^−3^. Finally, an AlGaAs shell was grown at 580 °C for undoped and Be-doped samples. Concerning Si-doped NWs, they consist of undoped GaAs cores and GaAs:Si shells grown at 430 °C (shell thickness: 80–100 nm and nominal doping concentration: 6 × 10^18^ cm^−3^). A thicker GaAs:Si shell was grown without additional AlGaAs passivation to unambiguously investigate the effect of Si doping in GaAs.

### Coexistence of ZB and WZ phases

**Fig. 1:**
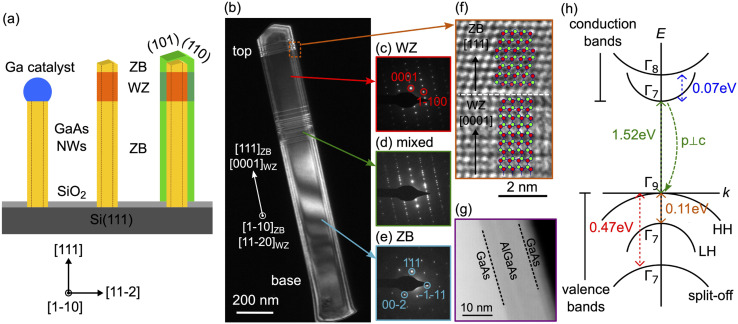
NW growth, crystal structure and WZ band diagram. (a) Schematics of the GaAs NW growth sequence on a patterned Si (111) substrate, starting from the GaAs stem grown using the self-catalyzed VLS method, followed by crystallization of Ga catalyst droplets, and the final AlGaAs shell overgrowth. (b) Dark field TEM image of a GaAs NW taken along the <1-10> zone axis, showing contrast between the ZB and WZ segments. (c–e) Local electron diffraction patterns showing the WZ/mixed phases/ZB structures along the NW height. (f) High-resolution HAADF STEM micrograph acquired at the top of the WZ section, showing the atomic details of the short ZB segment at the top end of the NW. (g) HAADF STEM micrograph taken along the <11-2> zone axis. The STEM contrast allows for the identification of the GaAs core, AlGaAs shell (∼13 nm) and GaAs outer shell (∼5 nm). (h) Band structure of bulk WZ GaAs. Band arrangement and approximate energy separation at the *Γ* point are given.

GaAs NWs grown with the self-catalyzed method usually have a ZB crystal structure, in contrast to Au-catalyzed growth.^5,50^ Indeed, we obtained pure ZB phase without twins over a length of 1 μm from the NW base, as seen in the dark field TEM micrographs and local electron diffraction patterns (Fig. 1(b–f)). A pure WZ segment (∼300 nm long) is found near the top region of the NWs and the crystal structure reverts back to ZB at the very top end. The occurrence of this WZ segment likely happened when the growth conditions were changed to obtain the crystallization of Ga droplets. Real-time TEM observations from the literature confirm that the crystal phase switching is often accompanied by the change of the droplet contact angle.^8,23,51^ A hexagonal prism usually develops at the crystallized NW tip, with facets of the {110} family.^52,53^ Finally, a NW “shell” was expanded *via* conventional vapor-solid growth to obtain a surface passivation with a higher band gap AlGaAs layer. The lateral overgrowth of the shell replicates the crystal structure of the core. Our NWs grown in the VLS step have typical core diameters of 90–110 nm. Hence, lateral quantum confinement is ruled out in this study. The choice of AlGaAs for the shell is to avoid lattice mismatch with the GaAs core, so that no additional strain is introduced and the presence of the shell does not modify the optical bandgap of the GaAs core. In Fig. 1(g), the High Angle Annular Dark Field Scanning TEM (HAADF-STEM) micrograph, taken along the [11-2] zone axis, shows the AlGaAs passivation layer of the shell (∼13 nm thick) and a thinner GaAs outer shell (∼5 nm thick) grown to prevent AlGaAs oxidation by air exposure.

### Band structure of WZ GaAs

A schematic band structure of bulk WZ GaAs is shown in Fig. 1(h), with the band ordering and approximate energy separations suggested in ref. 54. In the WZ structure, the valence band is split by spin–orbit coupling and by the hexagonal crystal field into three sub-bands: heavy hole (*Γ*_9V_), light hole (*Γ*_7V_) and split-off (*Γ*_7V_) valence bands. The conduction bands consist of one sub-band (*Γ*_7C_), which matches that of ZB, and another one (*Γ*_8C_) associated with a larger electron effective mass and arising from zone folding of the ZB L-valley. Small exciton reduced masses deduced from magneto-PL measurement (*μ*_‖_ = 0.057*m*_0_ and *μ*_⊥_ = 0.052*m*_0_, where *m*_0_ is the free electron mass),^54^ suggest that the lower conduction band should have *Γ*_7_ symmetry.^55^ Light emission involving *Γ*_8C_ is more difficult to observe because the oscillator strength of *Γ*_8C_ − *Γ*_9V_ is much smaller than that of *Γ*_7C_ − *Γ*_9V_.^38^ Symmetry considerations in the WZ lattice using group theory allow establishing the optical selection rules:^56^ emission from the *Γ*_7C_ − *Γ*_9V_ and *Γ*_8C_ − *Γ*_9V_ transitions are linearly polarized perpendicular to the *c*-axis; *Γ*_7C_ − *Γ*_7V_ is allowed in all directions; and *Γ*_8C_ − *Γ*_7V_ is forbidden.

## CL experiments

CL measurements were performed using an Attolight Allalin Chronos Quantitative Cathodoluminescence microscope. After the growth, NWs were dispersed on a Si host substrate. Single NWs were excited with a focused electron beam of 6 kV acceleration voltage and an impinging current of about 0.3 nA (continuous excitation). At 6 kV, the excitation volume in GaAs has a depth of about 70 nm and a diameter of 35 nm, and light was collected through an achromatic reflective objective.^46–48^ For polarization measurement, light passed through a linear polarizer before being focused at the entrance slit of a Horiba iHR320 spectrometer (grating 150 grooves per mm) and then recorded with an Andor Newton Si CCD camera (1024 × 256 pixels, with a pixel width of 26 μm and a spectral dispersion of 0.53 nm per pixel). The polarization response of the whole system is measured using a bulk ZB GaAs sample, which should emit unpolarized light. The polarization response is constant in the spectral range of interest (800–900 nm). For time-resolved measurement, a pulsed electron beam was controlled *via* a 355 nm laser with a repetition rate of 81.8 MHz (pulse width: ∼5 ps every 12 ns) and luminescence was recorded using a Hamamatsu streak camera C10910-05 with a photocathode S-20ER.

## Undoped GaAs NWs

### CL polarimetry

**Fig. 2:**
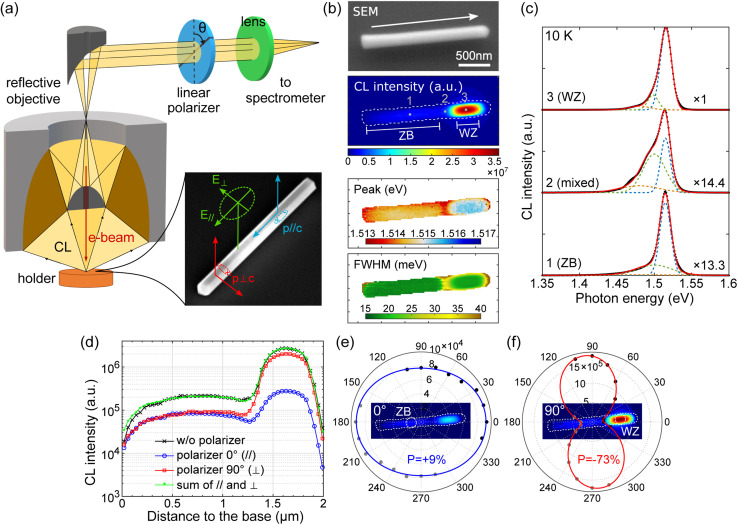
CL polarimetry measurement of WZ and ZB GaAs in an undoped nanowire at 10 K. (a) Schematics of CL polarimetry measurement. (b) Scanning electron microscope (SEM) image and CL maps (integrated CL intensity, CL peak position and FWHM) of an undoped GaAs NW acquired without a linear polarizer. The white arrow indicates the NW growth direction. (c) CL spectra extracted from the bottom 1 (ZB), 2 (mixed) and top 3 (WZ) regions of the NW, as marked in (b). The spectra are fitted with three Gaussians (colored lines), to separate the defect-related luminescence at the lower energy side from the main emission peak near 1.52 eV. (d) CL intensity along the NW measured without a polarizer (black) and with a linear polarizer oriented at 0° (blue, parallel to the NW) and 90° (red, perpendicular to the NW). (e and f) Polar plot of the collected CL intensity extracted from: (e) pure ZB and (f) pure WZ regions of the NW as a function of the polarizer angle. Insets show two CL intensity maps acquired under 0° and 90° polarizer angles. The degree of linear polarization is marked as *P*.

To investigate the NW properties, CL polarimetry measurements are performed at a low temperature of 10 K. Fig. 2(a) shows a schematic of electron excitation and light emission from a NW and the light collection path from the CL microscope. The continuous or pulsed electron beam excitation allows the local injection of electron–hole pairs. Subsequently, carriers may diffuse depending on the material quality, its band structure and doping. Radiative recombinations occur with electric dipoles oriented randomly or preferentially along certain directions according to the selection rules in the crystal lattice. Finally, light emission is mediated by the NW geometry, where the antenna effect may influence the polarization and emission direction of light.^57,58^ Here, we use a linear polarizer in front of the spectrometer to measure the degree of linear polarization of the collected light in order to distinguish the emission from the ZB and the WZ regions, which are spatially close in a single NW. NWs are dispersed on a Si substrate, so they lie flat on the imaging plane with random orientations.

Fig. 2(b) shows the SEM image of an undoped GaAs NW and the corresponding CL maps: the CL intensity integrated over the full spectral range, the peak position and the full width at half maximum (FWHM). The CL spectra extracted from the positions 1, 2, and 3 indicated in the CL intensity map are presented in Fig. 2(c). From the ZB region, the CL peak around 1.514–1.515 eV (FWHM: 17 meV) is typical of a free exciton and an unresolved bound exciton in ZB GaAs. In the WZ region, the CL peak is blue-shifted by 1 meV (FWHM: 19 meV) with respect to the ZB region. Defect-related emission at lower energy is more visible in the mixed region between the pure ZB/WZ phases (shoulder around 1.49 eV).

To confirm the spatial origin of the CL emission, we analyze the CL linear polarization. Fig. 2(d) plots the CL intensity along the NW measured without a polarizer (black) and with a polarizer parallel (blue) and perpendicular (red) to the NW axis. The green curve represents the sum of the two orthogonal measurements and coincides with the black curve. The degree of linear polarization *P* is defined as1
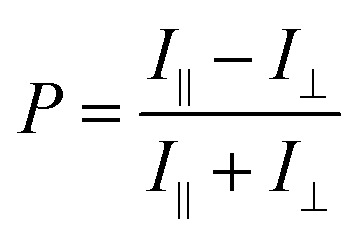
where *I*_‖_ (resp. *I*_⊥_) is the component of light intensity polarized parallel (resp. perpendicular) to the NW axis. The degree of linear polarization is corrected for the polarization response of the setup. Fig. 2(e) and (f) show the polar plots of CL intensities extracted from the ZB and WZ parts, respectively. The degree of polarization is fitted with the angular dependence of intensity using *I*(*θ*) = *I*_‖_ cos^2^ *θ* + *I*_⊥_ sin^2^ *θ*. The emission from the ZB GaAs presents a small degree of polarization (9%) parallel to the NW axis, which can be explained by the dielectric mismatch between the NW and the surrounding environment,^41,59^ while the CL emission from the WZ GaAs shows a remarkable degree of polarization of 73% with an emission oriented perpendicularly to the NW axis. This polarization anisotropy allows ruling out carrier diffusion far from the mixed ZB/WZ region. The ∼1.516 eV emission peak can be attributed unambiguously to the exciton recombination in WZ GaAs.

CL emission at lower energy (shoulder around 1.49 eV) is more visible in the mixed region between the pure ZB/WZ phases (Fig. 2(c)) and may be related to shallow defects. The ZB/WZ heterostructure might also lead to redshifted emission due to spatially indirect recombination in type-II band alignment.^37^ In ZB/WZ GaAs, electrons are preferentially relaxed to the ZB part with lower energy, while holes drift to the WZ part. A band offset around 115 meV was deduced from a fit of PL data of ZB insertions in WZ NWs at low excitation power.^60^ We observe no evidence of spatial localization of the low-energy emission, and the emission peak is found independent of the injection level (see the ESI†), suggesting that they are more likely due to impurities or native defects present in NWs.

### Time-resolved CL

**Fig. 3:**
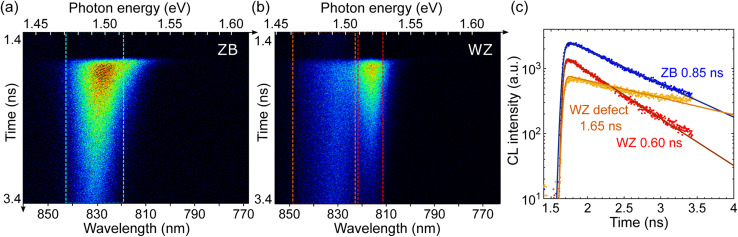
Time-resolved CL measurement of an undoped GaAs NW. (a and b) Streak images of the luminescence decay acquired when the pulsed electron beam excites: (a) the ZB and (b) the WZ part of the NW. (c) Time decay of the luminescence integrated over the different spectral ranges: (blue) ZB 819–843 nm (1.471–1.514 eV), (red) WZ 812–822 nm (1.509–1.527 eV), and (orange) WZ 823–849 nm (1.461–1.507 eV).

Carrier lifetimes are characterized using TRCL measurements. The use of a pulsed electron beam can reveal the dynamics of carrier transport and recombination at the nanoscale.^61,62^ Here, experiments are carried out at 10 K, with an electron pulse width of 5–10 ps every 12 ns, and the linear polarizer is used to distinguish the WZ and ZB phases. We observe clear exponential decays with lifetimes of the order of 1 ns. Fig. 3(a) and (b) show the images acquired with a streak camera over 2 ns time windows when the electron beam excites the ZB and the WZ parts of an undoped GaAs NW, respectively. Low-energy emission is more visible due to the much lower excitation current in pulsed mode, about three orders of magnitude lower than with continuous excitation (see the ESI†). In the ZB GaAs phase, the small redshift of the CL peak observed along the decay is consistent with the effect of lower carrier concentration, as shown under continuous excitation in Fig. S1(b).† However, we emphasize that the ZB defect band below 1.5 eV is only seen at very low carrier densities obtained under pulsed excitation and not under our continuous injection conditions where excitonic emission dominates. The ZB CL peak at 1.494 eV is likely due to recombination (e,A), involving free electrons and a C acceptor. Carbon residue from the MBE growth may also be present in the WZ segments. Therefore, the ∼1.52 eV transition can be attributed to the free exciton and 1.49–1.50 eV corresponds to the (e,A) transition in WZ GaAs.

In Fig. 3(c), the decays of these three transitions are compared. Excitons dominate the initial stage of radiative recombination in WZ GaAs, with an exciton lifetime of 0.6 ns. The free-to-bound defect luminescence takes place with a longer decay of over 1 ns. The luminescence decay in ZB exhibits a lifetime of 0.85 ns. Lifetimes of the order of ns are comparable with the literature^63,64^ and demonstrate effective electronic passivation from the higher band gap AlGaAs shell.

## Be-doped GaAs nanowires

In the following, we investigate the incorporation of dopants into WZ GaAs. Two p-doped NW samples are grown under the same VLS conditions as the previous undoped GaAs NWs, but with an additional Be flux. The doping concentration in the ZB part of the NWs of these two samples was previously characterized by CL analysis, and a quantitative determination of the hole concentration was demonstrated using the bandgap narrowing effect,^47,48^ resulting in doping concentrations of 2.3–3.3 × 10^18^ cm^−3^ (p-doped NWs) and 1.0–1.2 × 10^19^ cm^−3^ (p^+^-doped NWs). Here, we use CL polarimetry to probe these two samples and to unveil the effect of Be doping in the WZ part.

**Fig. 4:**
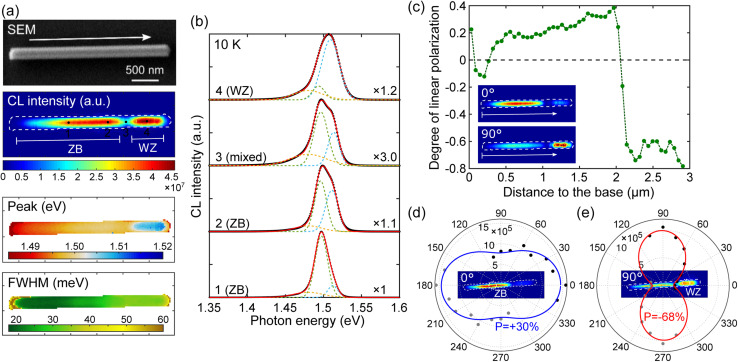
CL measurement of a Be-doped GaAs NW. (a) SEM and CL maps (integrated CL intensity, CL peak position and FWHM) of a Be-doped GaAs NW. The arrow indicates the NW growth direction. (b) CL spectra extracted along the NW (labeled 1–4 in the CL intensity map) and fits with three Gaussian (colored lines). (c) Degree of linear polarization along the NW deduced using two CL measurements with 0° and 90° polarizer angles (insets show the integrated intensity maps). (d and e) Polar plot of CL intensities of another NW of the same sample measured under different polarizer angles. CL intensities are extracted from (d) the ZB part and (e) the WZ part.

Fig. 4(a) shows the SEM image and CL maps of the p-doped GaAs NW (2.3–3.3 × 10^18^ cm^−3^) recorded at 10 K. The spectra extracted along the NW length (labeled 1–4) are presented in Fig. 4(b). Similar to the undoped GaAs NWs containing a long ZB base and a short WZ segment, CL maps of the Be-doped NW also present two distinct sections with slightly different characteristics. The polarization anisotropy is resolved, with the degree of linear polarization along the NW plotted in Fig. 4(c) (insets show the integrated CL intensity maps recorded with the polarizer parallel (0°) or perpendicular (90°) to the NW). Fig. 4(d) and (e) show polar plots of CL intensities extracted from the ZB and WZ parts, respectively, and confirm the existence of WZ segments in Be-doped NWs without systematic TEM studies. Again, the sharp contrast observed in the profile of polarization suggests that the carrier diffusion between ZB/WZ sections is minor at low temperature, allowing the properties of Be-doped WZ GaAs to be probed by CL.

**Fig. 5:**
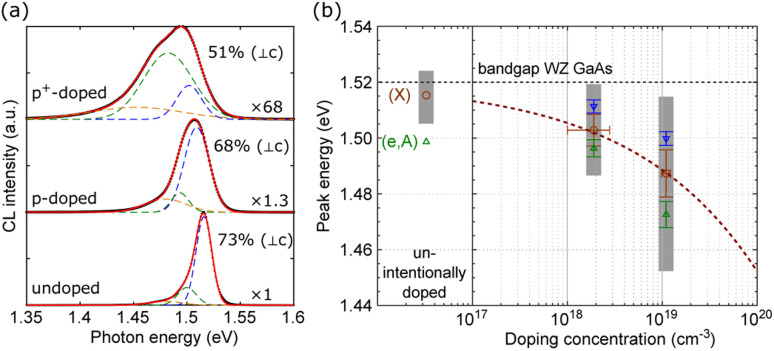
Comparison of undoped/Be-doped WZ-GaAs. (a) CL spectra extracted from the top (WZ) part of undoped and two Be-doped GaAs NWs. The measured degrees of linear polarization perpendicular to the *c*-axis are indicated. (b) CL peak energies as a function of the nominal doping concentration p. Circles mark the peak energy and triangles indicate eventual shoulders obtained from three-Gaussian fits. Gray bars represent the FWHM of each CL spectrum. The dashed line is an empirical fit of the peak energy with a *p*^1/3^ function.

Similar CL measurements on a p^+^-doped GaAs NW (1.0–1.2 × 10^19^ cm^−3^) are provided in the ESI.† In Fig. 5(a), we compare the CL spectra of undoped, p-doped and p^+^-doped WZ GaAs. With increasing Be doping concentrations, WZ GaAs exhibits redshifted and broadened CL lineshapes similarly to ZB GaAs, but with a degree of polarization over 50% and perpendicular to the NW axis. Indeed, in acceptors with a fully occupied s atomic shell, the optical transition between *Γ*_7C_ and the hole bound states originating from unoccupied p orbitals is polarized perpendicular to the *c*-axis.^65^ The degree of polarization decreases slightly with increasing doping concentration. This can be regarded as an increase of disorder due to doping so the optical selection rule is relaxed. In Fig. 5(b), we plot the CL characteristics of the three samples (peak energy and FWHM) as a function of the nominal doping concentration calculated from the CL data of the GaAs ZB phase. This assignment of doping concentration in WZ GaAs is only indicative. Circles and bars indicate the CL peak positions of WZ GaAs and typical variations observed. Upper and lower triangles represent different peaks/shoulders in the CL spectra. Grey bars mark the positions at half-width of the whole CL spectra. The low-energy shoulder seen for the p-doped sample near 1.49 eV may be attributed to the (e,A) transition in WZ GaAs involving the Be acceptor (ionization energy: ∼30 meV).^66,67^ Above 10^18^ cm^−3^, the acceptor band overlaps with the valence band and CL spectra account for the overall bandgap narrowing. The dashed line is an empirical fit for WZ GaAs, where *p* is the doping concentration in cm^−3^:

2 CL peak (shift, eV) = 1.5 × 10^−8^ × *p*^1/3^.

## Si-doped GaAs nanowires

**Fig. 6:**
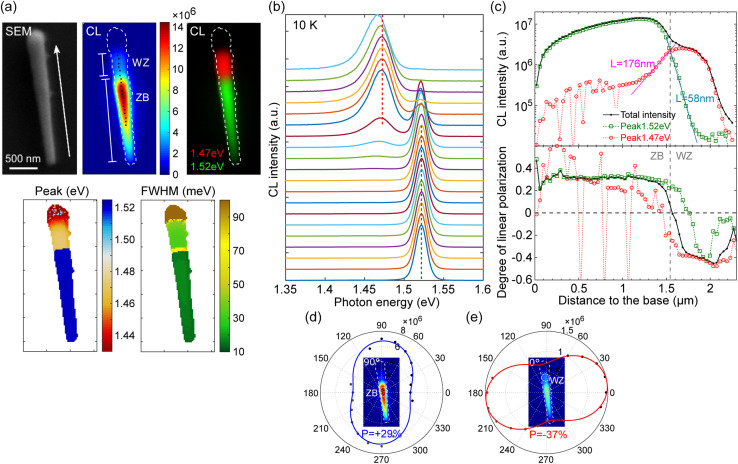
CL measurement of a Si-doped GaAs NW. (a) SEM and CL maps (integrated CL intensity, panchromatic CL intensity at 1.47 eV and 1.52 eV, CL peak position and FWHM). (b) CL spectra (normalized) extracted along the NW. Green and red dashed lines indicate 1.52 eV and 1.47 eV, respectively. (c) CL intensity and degree of linear polarization along the NW obtained by regarding the total intensity (black), intensity of the 1.52 eV peak (green), and intensity of the 1.47 eV peak (red). (d and e) Polar plot of CL intensities acquired under different polarizer angles. CL intensities are extracted from (d) the ZB part and (e) the WZ part.

We now investigate n-type Si-doped WZ GaAs. Direct doping using Si during the VLS growth of GaAs NWs at high temperature (over 600 °C) was reported to cause p-type doping^68^. Here, we first grew undoped GaAs NW cores at 610 °C (diameter: 200 nm) and subsequently GaAs:Si-doped shells (thickness: 90 nm) at 480 °C to prevent the competing compensation of Si dopants in GaAs cores grown by self-catalyzed VLS.^46,48^Fig. 6(a) shows the SEM image of a Si-doped GaAs NW and the corresponding CL maps (integrated CL intensity, two-color CL maps with spectral bands at 1.47 eV and 1.52 eV, peak position and FWHM). CL spectra extracted along the NW are plotted in Fig. 6(b). The tip has very low CL intensity and corresponds to the shell overgrowth upon the top inclined facets of the core, which may present extended structural defects and is disregarded in the analysis. The attribution of the WZ segment is confirmed using CL polarimetry. In Fig. 6(c), we plot the total CL intensity along the NW (dark curve) together with the deconvolution of intensities of the 1.52 eV peak attributed to ZB GaAs in green and the 1.47 eV peak attributed to WZ GaAs in red. Total intensity from the ZB part shows *P* = 29% parallel to the NW axis, while the degree of polarization of the WZ segment is *P* = 37% perpendicular to the NW axis (Fig. 6(d) and (e)). The 1.52 eV peak (FWHM 22–26 meV) from the ZB part corresponds to the electron filling of 5 × 10^17^ cm^−3^ concentration.^48^ This signal decreases along the NW axis when the electron beam excitation moves into the WZ part, following an exponential decay exp(−*x*/*L*) with *L* = 58 nm corresponding to the carrier diffusion length in WZ GaAs. On the other hand, the peak at 1.47 eV is localized in the WZ segment and decreases exponentially into the ZB part with *L* = 176 nm corresponding to the diffusion length in ZB GaAs. The diffusion mechanism is also illustrated by the slow transition between ZB and WZ in the profile of the degree of polarization in Fig. 6(c).

A remaining question is the origin of the peak at 1.47 eV. It is observed only with Si doping and is still polarized perpendicular to the NW axis. Hence, it should be related to Si impurities embedded in the WZ lattice. Si atoms may occupy both Ga and As sites, forming donor–acceptor pairs or inducing other defect complexes. The degree of polarization lower than that of the exciton and (e,A) transitions observed previously may indicate a stronger localization of donor–acceptor pairs that suppresses the polarization anisotropy.^69^

## Conclusion

We have studied GaAs NWs containing large sections of ZB and WZ phases. TEM characterization revealed a long (>1 μm) ZB base without detectable twins and WZ top segments (∼300 nm) without detectable structural defects. CL polarimetry was used to distinguish the luminescence emitted by each segment in undoped, Be-doped and Si-doped GaAs nanowires, in order to investigate dopant incorporation into the WZ phase.

In undoped WZ GaAs, a peak at 1.516 eV was observed at 10 K with a degree of polarization of 73% perpendicular to the *c*-axis. It is attributed to the *Γ*_7C_ − *Γ*_9V_ exciton transition, in agreement with the bandgap of WZ GaAs being only slightly higher than that of ZB GaAs. From Be-doped samples, we inferred an ionization energy of ∼30 meV for Be acceptors in WZ GaAs. We find a quantitative bandgap narrowing and a decrease in the polarization degree with increasing hole concentration in WZ GaAs. On the other hand, Si-doped GaAs shells grown on the side wall facets of WZ GaAs resulted in a broad emission peak around 1.47 eV, characterized by a lower degree of polarization but still perpendicular to the *c*-axis. It suggests that Si impurity compensation or inherent defects are present in Si-doped WZ GaAs, forming active donor–acceptor pairs at low temperature.

The luminescence decay was also measured in the ZB and WZ segments of a single undoped nanowire by time-resolved cathodoluminescence, and carrier lifetimes between 0.65 ns (WZ exciton) and 1.65 ns (WZ shallow defect) were found at 10 K. These results, based on the combination of polarimetric and time-resolved CL to distinguish the different phases in a single nanowire, open the door to in-depth investigations in various semiconductor nanostructures.

## Data availability

Additional data supporting this article have been included as part of the ESI.†

## Conflicts of interest

There are no conflicts to declare.

## Supplementary Material

NA-007-D5NA00206K-s001
