# Supplementary material for: Undoped and doped wurtzite GaAs probed by polarization- and time-resolved cathodoluminescence
Source: Nanoscale Adv. 2025 Apr 10;7(11):3387–95. doi: 10.1039/d5na00206k (PMC12004390; doi:10.1039/d5na00206k)
Supplement: NA-007-D5NA00206K-s001 [file NA-007-D5NA00206K-s001.pdf]

Supplementary Information (SI) for Nanoscale Advances.
This journal is © The Royal Society of Chemistry 2025

Undoped and Doped Wurtzite GaAs Probed by Polarization- and Time-Resolved Cathodoluminescence: Supplementary Information

Hung-Ling Chen[1], Thomas Bidaud[1], Andrea Scaccabarozzi[1,2], Romaric De Lépinau[1,2], Fabrice Oehler[1], Gilles Patriarche[1], Sophie Bouchoule[1], Jean-Christophe Harmand[1], Andrea Cattoni[1], and Stéphane Collin[*1,2]

[1]Centre de Nanosciences et de Nanotechnologies (C2N), CNRS, Université Paris-Saclay, Palaiseau, France
[2]Institut Photovoltaïque d'Ile-de-France (IPVF), Palaiseau, France

[*]Corresponding author: stephane.collin@cnrs.fr

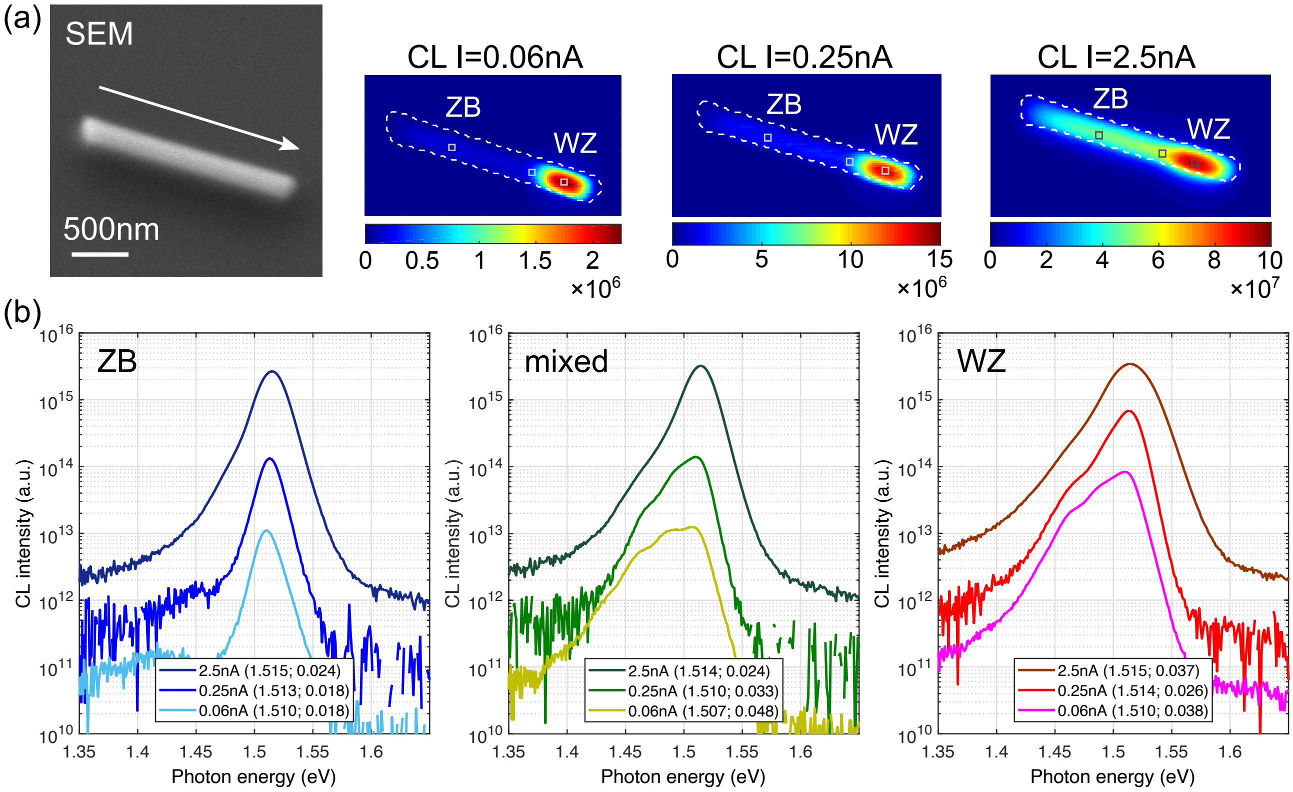

Figure S1: CL measurement of an undoped GaAs NW at different excitation powers. (a) SEM image of the NW and the integrated CL intensity maps for excitation current of 0.06 nA, 0.25 nA and 2.5 nA. (b) Evolution of the CL spectra extracted from different regions of the NW (ZB, mixed and WZ part).

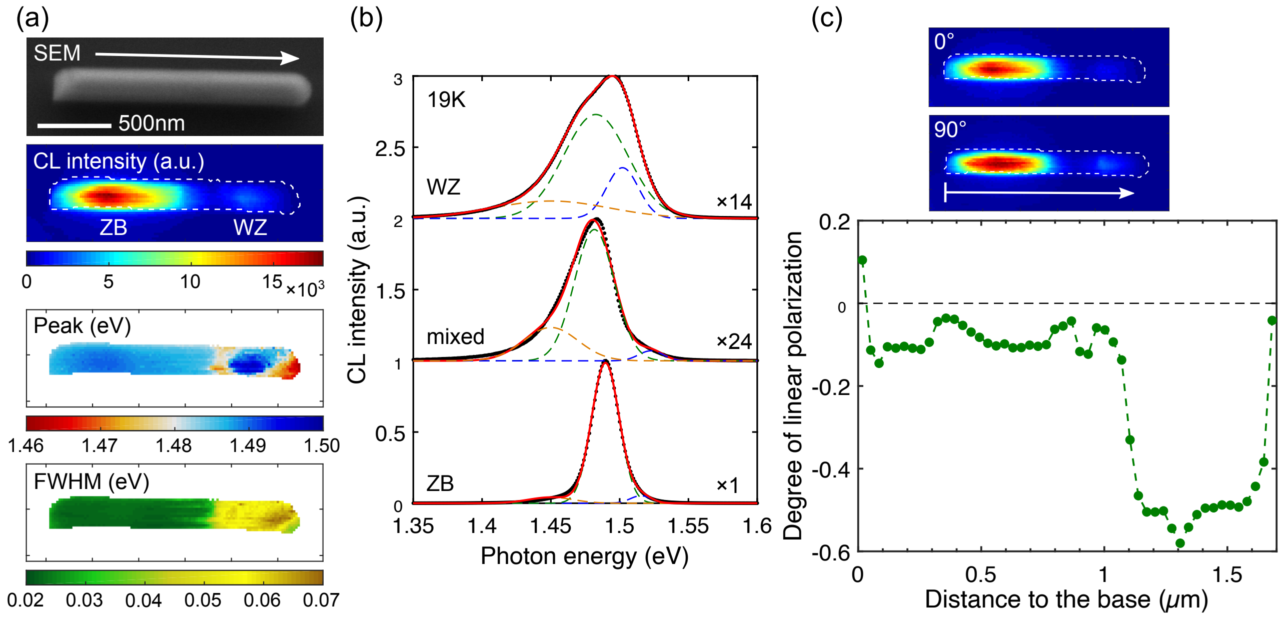

Figure S2: CL measurement of a p$^+$-doped GaAs NW. (a) SEM image and CL maps (integrated CL intensity, peak position energy and FWHM). (b) Comparison of CL spectra extracted for the ZB, mixed and WZ part of the NW (black dots). They are fitted using three Gaussians (dashed lines, red line: sum). (c) CL intensity maps measured with a linear polarizer parallel (0°) and perpendicular (90°) to the NW axis and the degree of linear polarization deduced along the NW.
